# A novel perspective on MOL-PCR optimization and MAGPIX analysis of in-house multiplex foodborne pathogens detection assay

**DOI:** 10.1038/s41598-019-40035-5

**Published:** 2019-02-25

**Authors:** Nikol Reslova, Veronika Huvarova, Jakub Hrdy, Martin Kasny, Petr Kralik

**Affiliations:** 1 Veterinary Research Institute, Department of Food and Feed Safety, Hudcova 296/70, 621 00 Brno, Czech Republic; 2 Faculty of Science, Department of Botany and Zoology, Masaryk University, Kotlářská 2, 611 37 Brno, Czech Republic; 3 Faculty of Science, Department of Experimental Biology, Masaryk University, Kamenice 753/5, 625 00 Brno, Czech Republic

## Abstract

Multiplex oligonucleotide ligation-PCR (MOL-PCR) is a rapid method for simultaneous detection of multiple molecular markers within a single reaction. MOL-PCR is increasingly employed in microbial detection assays, where its ability to facilitate identification and further characterization via simple analysis is of great benefit and significantly simplifies routine diagnostics. When adapted to microsphere suspension arrays on a MAGPIX reader, MOL-PCR has the potential to outperform standard nucleic acid-based diagnostic assays. This study represents the guideline towards in-house MOL-PCR assay optimization using the example of foodborne pathogens (bacteria and parasites) with an emphasis on the appropriate choice of crucial parameters. The optimized protocol focused on specific sequence detection utilizes the fluorescent reporter BODIPY-TMRX and self-coupled magnetic microspheres and allows for a smooth and brisk workflow which should serve as a guide for the development of MOL-PCR assays intended for pathogen detection.

## Introduction

Foodborne pathogens (FBP) represent one of the major threats to public health, especially in developing countries^1^, and thus, food safety remains one of the most important global health issues. Foodborne illnesses are usually related to pathogenic viruses, bacteria, and parasites as well as toxic chemical substances entering the body through contaminated food or water^2^. Similar clinical manifestations may have at their root various causes, and, therefore, a complex and effective system of prevention is required; in this process, the timely detection and further characterization of pathogens plays a critical role^3^. Nucleic acid-based assays are ideal for such purposes due to their high specificity, ability to be modified to detect almost any target, minimal sample amounts required (high sensitivity), the short time required for analysis, and the capability for automation^4^. Such assays should also enable simultaneous screening of numerous molecular markers, which would be time- and cost-effective in comparison to gold standard methods, such as, e.g. cultivation, immunoassays, biochemical tests, and PCR/qPCR that require multiple serial or parallel assays and have limitations in multiplexing ability^3,5^.

The multiplex oligonucleotide ligation-PCR (MOL-PCR) first described by Deshpande *et al*.^4^ combines analysis of multiple types of molecular markers in a single multiplex reaction^4,6,7^ and therefore is capable of specific sequence detection in parallel with SNP identification or detection of indels (insertion/deletion). Owing to these features, MOL-PCR represents a sensitive tool for complex screening of pathogens (virus, bacteria, fungi) in various matrices or to establish the presence of genes responsible for antibiotic resistance. The visualization of MOL-PCR products can be achieved using several detection platforms, e.g. fixed microarrays, gel-based formats, or microsphere suspension arrays^4^. The latter way of visualization using a Luminex MAGPIX (Luminex Corp., Texas, USA) or Bio-Plex MAGPIX Multiplex Reader (Bio-Rad, California, USA), hereafter referred to as MAGPIX, allows for simple workflow and reproducible results. The MAGPIX is a simple instrument based on a suspension array using magnetic microspheres. The instrument consists of a magnet for immobilization of magnetic microspheres and two diodes that read the fluorescent spectra of dyes within the microspheres and reporter molecules captured on their surface^8^. Moreover, MAGPIX has the ability to analyze up to 50 markers (limited by the capacity of regions for magnetic microspheres) in a single sample.

**Figure 1:**
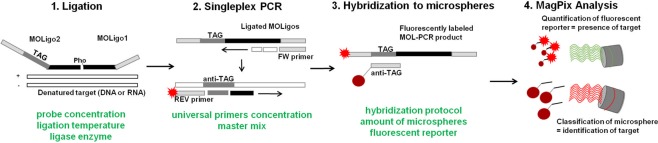
Overview of MOL-PCR assay workflow. The workflow is illustrated for one target. Green headings indicate several parameters of the MOL-PCR reaction, which must be optimized in order to achieve the best results. White = target DNA. Black = complementary region of specific detection probes. Gray = universal primer sites. Red stain = fluorescent reporter.

Carrying out of a typical MOL-PCR assay involves three crucial steps: multiplex oligonucleotide ligation of specific probes for detection of molecular markers, PCR for signal amplification, and the hybridization of PCR products to magnetic microspheres followed by signal detection on MAGPIX (Fig. 1). The advantages of MOL-PCR lie in the multiplex ligation step, which is carried out prior to the PCR amplification and in the fact that specific detection probes are modular structures bearing binding sequences for universal primers enabling singleplex PCR^9^. Compared to other ligation-based methodologies such as multiplex ligation-dependent probe amplification (MLPA) or the oligonucleotide ligation assay (OLA), where multiplexed PCR is the limiting factor of high multiplexing (due to different lengths of the amplicons), in MOL-PCR the ligation step represents the actual detection event and the subsequent PCR does not serve for amplification of template DNA^10^. This ensures that MOL-PCR is not susceptible to amplification bias or cross-hybridization, which are characteristic not only of other ligation-based assays but also of multiplex PCR^8,11^.

Modular detection probes used in MOL-PCR, in particular MOLigo1, consist of two segments: a target-complementary sequence at the 5′-end followed by a reverse-complement of the universal FW primer sequence; importantly, the 5′-end of MOLigo1 is phosphorylated to enable ligation to MOLigo2. The longer MOLigo2 contains three segments: a universal REV primer sequence at the 5′-end linked to an inner specific 24-bp xTAG sequence (synthetic sequences based on just three bases T, A, and G; provided by Luminex Corp., Texas, USA) and target-complementary sequence at the 3′-end. If hybridization of probes to their target sequence occurs, DNA ligase links them covalently into one complex sequence (100–120 nucleotides long). Ligated sequence serves as a template for singleplex PCR, where the REV primer carries a fluorescent reporter (depicted as red stain in Fig. 1) marking the product. MOL-PCR products are hybridized to the anti-TAG sequences on the surface of magnetic microspheres and detected in MAGPIX.

Although the popularity of MOL-PCR is increasing, only little is found in the literature regarding the optimization steps which must be implemented before finalization of the working protocol. However, home-made assays intended for routine use need to be optimized in terms of differentiation between positive and negative results, cost, and effort. To date, relevant papers have focused mainly on detection of single nucleotide polymorphisms (SNPs)^6,10,12^, have utilized indirect labeling by the fluorescent dye streptavidin-R-phycoerythrin (SAPE)^5–7,13^, and were adapted for measurement on the Luminex 100/200 (Luminex Corp., Texas, USA) flow cytometer device^4,6,10,12^. Further, in all cases commercially pre-coupled MagPlex-TAG Microspheres (Luminex Corp., Texas, USA) were used. While these papers did describe the reaction conditions in detail, they nevertheless failed to provide a comprehensive description of the optimization procedure. Wuyts *et al*.^7^ created guide of optimization focused on MagPlex-TAG Microspheres and following a slightly adapted workflow of the commercially available manufacturer’s Cookbook protocol^14^. However, a precise and detailed description of optimization experiments together with troubleshooting is crucial and essential for the development of any in-house MOL-PCR assay for pathogen detection. Therefore, the present study was meant to be a guideline through such MOL-PCR optimization and was aimed at identifying and analyzing in detail individual parameters of the working protocol on two model organisms. These parameters, may, if inappropriately chosen, negatively affect the workflow and performance of the whole analysis. The final optimized conditions were tested in an 11-plex model assay targeting a set of bacterial and parasitic pathogens transmitted via food matrices. In addition, in this study, we tried as much as possible to fulfill criteria with respect to the availability of reagents and equipment, affordability, and the skills required to perform the method, with the aim of making this approach feasible for a wide range of users who carry out detection and identification of pathogens in food.

## Materials and Methods

### Target organisms and DNA template

The most important parameters in the development of a MOL-PCR assay designed for detection of pathogens intended for routine application were optimized in two model organisms, the foodborne pathogens *Yersinia enterocolitica* (*YE*) and *Toxoplasma gondii* (*TG*).

The *YE* CAPM 6154 bacterial isolate (serotype O2,3; biotype 3) was obtained from the Collection of Animal Pathogenic Microorganisms at the Veterinary Research Institute (Brno, Czech Republic). A single colony from a culture was resuspended in dH_2_O and genomic DNA (gDNA) was released by application of heat lysis at 100 °C for 15 min. After centrifugation, 50 μl of supernatant were diluted in 450 μl of dH_2_O (10X dilution).

*TG* oocysts obtained from the National Reference Laboratory for Parasites at the University of Veterinary and Pharmaceutical Sciences (Brno, Czech Republic) were used for gDNA isolation using an extraction protocol previously described in Reslova *et al*.^15^. Briefly, 10 μl of oocysts in H_2_O (approximately 2 × 10^6^ oocysts) were incubated in 200 μl of extraction buffer (100 mM Tris-Cl from Sigma-Aldrich, Missouri, USA; 10 mM EDTA from Amresco, Ohio, USA; 100 mM NaCl from Carl Roth, Germany; 1% SDS from Sigma-Aldrich, Missouri, USA; 1.5 mM dithiothreitol from Roche, Switzerland and 0.06 mg proteinase K from Qiagen, Germany) overnight at 55 °C. Proteins were precipitated using 3 M sodium acetate and gDNA-containing supernatant was precipitated by ice-cold 99.5% isopropanol. After incubation at −70 °C for 30 min and centrifugation the pellet was washed using 200 μl of 70% ethanol. Finally, pelleted gDNA was dissolved in 50 μl of dH_2_O and diluted to a concentration of 1 ng/μl. Both gDNAs were stored at −20 °C until further use.

### Probe design

Specific MOLigos were targeted to the one-copy *ail* gene, which encodes an outer membrane protein and a multi-copy 529-bp repeat element, for the detection of *YE* and *TG*, respectively. These targets were chosen with regard to their frequent and validated use in detection methods and the availability of their sequences in the NCBI GenBank database. Then, an approximately 100-bp long region was chosen manually and tested in OligoAnalyzer 3.1 (https://eu.idtdna.com/calc/analyzer) with the aim of finding the most suitable region for probe hybridization. The complementary region of each probe should meet certain parameters, as described in detail in Deshpande *et al*.^4^. The melting temperature of target complementary regions was set to be at least 47 °C, which is high enough to ensure detection specificity^4^. The optimal melting temperatures of designed MOLigos has been suggested to be in the range 47–58 °C with an average of 53 °C in most probes. Moreover, the difference in melting temperatures between the two complementary regions of one MOLigo pair did not exceed 1.5 °C. Sequences with hairpin melting temperatures higher than 30 °C and sequences for which potential dimer formation (dG < −7 kcal/mol) was identified were excluded. After setting the parameters, complementary sequences were checked in Nucleotide BLAST to guarantee their uniqueness for the desired target and to ensure that probe efficiency would not be reduced by off-target interactions. MOLigo probes were synthesized using standard desalting purification (Generi-Biotech, Czech Republic; Table 1).

**Table 1:** Sequences for primers and MOLigo pairs.

Oligo type	xTAG	Sequence 5′-3′
TG_MOLigo1		Pho-CGGAAACATCTTCTCCCTCTCC**TCTCACTTCTTACTACCGCG**
TG_MOLigo2	A018	*ACTCGTAGGGAATAAACCGT*GtaattgaattgaaagataagtgtTCCAGGAAAAGCAGCCAAGC
YE_MOLigo1		Pho-GGAGTAATAGGTTCGTTTG**TCTCACTTCTTACTACCGCG**
YE_ MOLigo2	A019	*ACTCGTAGGGAATAAACCGT*gtgtgttatttgtttgtaaagtatGAACTCGATGATAACTGG
Universal FW primer		**CGCGGTAGTAAGAAGTGAGA**
Universal REV primer		*ACTCGTAGGGAATAAACCGT

Target-hybridizing complementary sequence is underlined, universal forward primer in bold, universal reverse primer in italics, and xTAG sequence is differentiated by lowercase. Pho = phosphorylation, *Fluorescent label.

### Coating of microspheres

MagPlex Microspheres (12.5 × 10^6^ microspheres/ml; Luminex Corp., Texas, USA) were coupled with anti-TAG sequences according to an adapted Bio-Plex Bead Coupling protocol by Bio-Rad, California, USA.

Magnetic microspheres of regions 19 and 34 were tempered to room temperature, vortexed on max speed, and sonicated for 30 s in an Ultrasonic Cleaning Bath (BioTech, Czech Republic). Then, 400 μl of each set were transferred into separate DNA LoBind Tubes (Eppendorf, Germany) and placed in the DynaMag-2 magnetic separator (Thermo Fisher Scientific, Massachusetts, USA) for 1 min in order to remove the supernatant. The pellet was resuspended in 45 μl of 0.1 M MES buffer, pH 4.5 (Sigma-Aldrich, Missouri, USA) and then vortexed and sonicated. Coupling reactions were started by the addition of 2 μl of 100 μM (stock in dH_2_O) A018 and A019 anti-TAGs (complementary to xTAG sequences in MOLigo probes in Table 1) with C6-amino modifications at the 5′-end; each anti-TAG was transferred into a separate tube with different microspheres and vortexed. Then, 2.5 μl of a 10 mg/ml freshly prepared solution of EDAC powder (Thermo Fisher Scientific, Massachusetts, USA) were added and the mixture was vortexed immediately. Tubes were incubated for 30 min in the dark at room temperature with vortexing every 10 min. Then, addition of 2.5 μl freshly prepared EDAC was repeated together with incubation and vortexing. After the coupling reaction, microspheres were washed in 1 ml 0.02% Tween 20 (Alpha Diagnostic, Texas, USA) by vortexing and the supernatant was removed using the magnetic separator. The pellet was resuspended in 1 ml 0.1% SDS, vortexed, and the supernatant was removed on the separator. Finally, the pellet was resuspended in 80 μl of 1X TE buffer (pH 8.0; SERVA, Germany), vortexed, sonicated, and the coated microspheres were stored in the dark at 4 °C where they remain stable and ready to use for more than one year^14^.

A hemocytometer was used to enumerate coupled microspheres (microspheres/μl). Microspheres were vortexed, diluted 1:100 in dH_2_O, and 10 μl were transferred to the hemocytometer, where four large corners of the grid were counted. The following simple formula was used for enumeration: microsphere concentration (microspheres/μl) = (sum of microspheres in four large corners) × 2.5 × 100 (dilution factor). The average concentration for both regions was 50,000 microspheres/μl (should be at least 40,000 microspheres/μl), which is sufficient for at least 1,600 reactions.

The quality of microsphere coating was verified by direct hybridization to xTAG oligonucleotides (A018 and A019, the same as the xTAG sequence in MOLigo2) fluorescently labeled at the 5′-end, which were then used in a concentration gradient of 0–100 femtomoles. These oligos simulated MOL-PCR products and the validation reaction was prepared in the same way as proper analysis according to the protocol described below (Hybridization to microspheres and MAGPIX analysis).

### Multiplex oligonucleotide ligation

**Figure 2:**
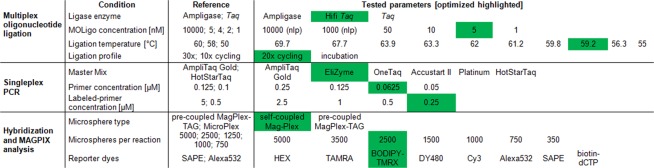
Table showing individual parameters of MOL-PCR assay which should be taken into account during optimizations. Reference values represent data reported in up-to-date literature (Deshpande *et al*.^4^; Thierry *et al*.^6^; Stucki *et al*.^10^; Wuyts *et al*.^5^; Woods *et al*.^13^) and optimized values are based on protocol described in present study. In green = significant values fullfilling interpretation criteria. (nlp) = no ligation product.

The multiplex ligation step was separated from PCR amplification^4^ in order to avoid the high background signal levels when combining both steps in one reaction^10^. For the optimizations, each ligation reaction was prepared in a singleplex manner and contained only one specific MOLigo pair and one gDNA template. In this set-up, various parameters of the ligation reaction were tested (Fig. 2: Multiplex oligonucleotide ligation) and all experiments were run in quadruplicates. The optimized ligation reaction mix combined 5 nM of each MOLigo probe (Table 1) with 2.5 μl of 10X Hifi *Taq* DNA ligase reaction buffer, 0.5 μl of Hifi *Taq* DNA ligase (New England BioLabs, Massachusetts, USA), and 2.5 μl of template DNA (corresponds to ∼2.5 ng of isolated DNA). The reaction was brought to a final volume of 25 μl with PCR H_2_O (Top-Bio, Czech Republic). The thermal cycling program (DNA Engine Dyad, Bio-Rad, California, USA) included 10 min of denaturation at 95 °C followed by 20 cycles of 30 s at 95 °C and 1 min at 60 °C. Reactions were then cooled to 10 °C and used immediately in the PCR amplification step. Each experiment consisted of a positive sample containing template DNA and a no-template-control (NTC) with PCR H_2_O instead of template to monitor cross-reactivity and contamination.

### Singleplex PCR

Different master mixes and concentrations of universal primers were tested (Fig. 2: Singleplex PCR).

The optimized singleplex PCR reaction was performed in a final volume of 24 μl, which included 12 μl of 2X EliZyme HS Robust MIX (Elisabeth Pharmacon, Czech Republic), 0.0625 µM of universal FW primer, 0.25 µM of BODIPY-TMRX-labeled REV primer (Table 1), and 6 μl of ligation product. The thermal cycling program consisted of initial denaturation at 95 °C for 2 min, followed by 40 cycles of 95 °C for 15 s, 60 °C for 15 s, and 72 °C for 15 s. Reactions were cooled to 10 °C and either used immediately in the microsphere hybridization step or stored at 4 °C pending hybridization.

Several master mixes were tested in the optimization experiments, namely AccuStart II PCR ToughMix (QuantaBio, Massachusetts, USA), AmpliTaq Gold 360 Master Mix (Thermo Fisher Scientific, Massachusetts, USA), One*Taq* Hot Start 2X Master Mix with GC Buffer (New England BioLabs, Massachusetts, USA), Platinum Hot Start PCR Master Mix (Invitrogen, California, USA), and HotStarTaq DNA Polymerase (Qiagen, Germany). Each singleplex PCR reaction and thermal cycling protocol was run according to the relevant instructions from the manufacturer. In order to increase SAPE signal, we tested addition of Biotin-16-dCTP (Jena Bioscience, Germany) to the PCR reaction mix in a ratio of 1:1 to the dCTPs included within the original EliZyme master mix.

### Hybridization to microspheres and MAGPIX analysis

The MOL-PCR products were hybridized to a particular magnetic microsphere set using xTAG sequences complementary to anti-TAG sequences covalently linked to the microspheres’ surface. All microspheres present in the reaction were then analyzed by a MAGPIX reader, whose red diode identified a specific microsphere set according to its spectral signature and whose green diode measured fluorescent intensity emitted by the fluorescent reporter bound to the captured analyte on the microsphere surface.

Altogether, three parameters of the hybridization step were tested (Fig. 2: Hybridization and MAGPIX analysis). An optimized microsphere mix consisting of self-coupled MagPlex microspheres of regions 19 and 34 specific to *YE* and *TG* MOLigo pairs contained 2,500 microspheres of each set, 800 mM NaCl, and 50 mM MES buffer; the volume of each reaction was adjusted to 5 μl with 1X TE buffer (pH 8.0). Ten microliters of MOL-PCR product were mixed with 5 μl of microsphere mix in 0.2 ml Tear-off Strips which were then closed with strip caps (Bioplastics, Netherlands). The hybridization reaction (based on an adjusted protocol by Deshpande *et al*.^4^) was performed in a thermal cycler and consisted of denaturation at 94 °C for 1 min, followed by a slow ramp down in temperature to 25 °C at a rate of 0.1 °C per second. Before analysis on a MAGPIX reader, the reaction volume was increased to 60 μl by adding 45 μl of analysis buffer containing 10 mM Tris-Cl (pH 8.0), 0.1 mM EDTA, 90 mM NaCl and 0.02% Tween 20. Strips were placed into a 0.2 ml Multo Rack bench (Bioplastics, Netherlands) and analyzed in MAGPIX device (Bio-Plex MAGPIX from Bio-Rad) using Bio-Plex Manager MP Software (Bio-Rad, California, USA).

When biotinylated REV primers or biotin-dCTPs were included during the optimization of the PCR step, the analysis buffer was enriched with SAPE (Thermo Fisher Scientific, Massachusetts, USA) in a final concentration of 3 μg/ml and with bovine serum albumin (BSA) in a final concentration of 0.1%. Samples were additionally incubated at 37 °C for 15 min. MAGPIX analysis was then carried out in the same manner but with heating at 37 °C.

Median fluorescence intensity (MFI) values were calculated from the analysis of at least 50 microspheres of each region per sample. After the analysis, the MFI values were exported from Bio-Plex Manager 6.1 Software (Bio-Rad, California, USA) into an Excel file to calculate signal-to-noise ratios.

### Multiplex assay

The functionality of the optimized protocol described above, including the probe design, coating of microspheres, multiplex oligonucleotide ligation, singleplex PCR, hybridization to microspheres and MAGPIX analysis, was demonstrated on a model multiplex assay developed in-house.

For the 11-plex panel, common representatives of foodborne pathogens were selected and five bacterial systems (*Y*.*enterocolitica; Escherischia coli* CAPM 5358, serotype O26, gene *wzy*; *Listeria monocytogenes* CAPM 5879, serotype 1/2b, gene *rnc*; *Campylobacter jejuni* CAPM 6316, serotype 10, gene *hipO;* and *Salmonella enterica* CAPM 5445, serotype Typhimurium, gene *ttr*C), five parasite systems (*T*. *gondii; Trichinella spiralis*, target *ITS1; Taenia saginata*, gene *cox1*; *Giardia intestinalis* generic for all assemblages, gene *ef1α;* and *Giardia intestinalis* assemblage A, gene *tpi*), and an internal amplification control (IAC) were designed. The IAC was designed as a non-competitive synthetic sequence based on two ancient DNA sequences^16^; specifically the mitochondrial DNA of two extinct species – the Tasmanian tiger (*Thylacinus cynocephalus*) and the giant moa (*Dinornis struthoides*). This synthetic sequence was cloned into a plasmid and served for the differentiation of truly negative and false negative (inhibited) samples^17^. Sequences of MOLigo probes included within this model multiplex assay are available upon request.

Genomic DNAs were extracted using the same procedure as described above with respect to bacterial or parasitic affiliation. All bacterial isolates were obtained from the Collection of Animal Pathogenic Microorganisms at the Veterinary Research Institute and single colony was used for heat lysis resulting in 10X diluted lysates. As an input into parasitic DNA isolation using extraction buffer was used muscle larvae of *T*. *spiralis* (provided by the International Trichinella Reference Center, Rome, Italy) and a part of a *T*. *saginata* body segment (originating from a natural infection). Genomic DNA of *G*. *intestinalis* was ordered commercially from ATCC, 30888D (Virginia, USA). DNA of each species was diluted to concentration of 1 ng/μl.

A single ligation reaction mix was prepared for all samples, which thus contained 22 specific MOLigo probes, each in final concentration 5 nM and 10^3^ of plasmid DNA as the template for the IAC. Four NTCs were prepared and the experiment was run in quadruplicates. For each sample one template DNA was used (∼2.5 ng) and microsphere mix consisted of 11 regions specific to individual pathogens.

### Data analysis and interpretation

For each experiment, two controls were used for the calculation of the signal-to-noise ratio: (1) A microsphere-only control called “blank” to report background fluorescence (contained analysis buffer, microsphere mix, fluorescent reporter, and dH_2_O instead of MOL-PCR product) and (2) a NTC to report cross-reactivity of MOLigos and contamination level in the absence of any template.

In an Excel file, all measured MFI values were corrected by subtraction of the blank MFI and then an average value for each sample/NTC was calculated from the quadruplicates. The signal-to-noise ratio was calculated by dividing the MFI of the sample by the corresponding MFI of the NTC. A signal-to-noise ratio of at least 4 and MFI of at least 200 were the two criteria used to determine “positive samples”^4^. Regarding the resulting data, it should be noted that even a slight increase in MFI values of NTCs as divisors can cause profound differences in the signal-to-noise ratios between samples.

Three criteria were considered for the comprehensive numerical evaluation of individual tested conditions for both targets *YE* and *TG*: MFI of the positive samples (MFI+), MFI of the NTCs (MFI-) and height of the signal-to-noise ratio (SNR). Each criterion has been given a different relevance (weight), corresponding to its importance during the data interpretation. In all parameters of the optimization, each tested condition was given a rating as a weighted mean of six ranks in individual criteria achieved in comparison test with other conditions. For each criteria, the following scale (expressing the importance) was chosen: MFI + 3 (high fluorescence intensity of positive samples is desirable, because a MFI decrease in the case of low DNA concentrations or high level of multiplex is expected), MFI- 2 (low fluorescence intensity of no-template controls is desirable but not so significant beyond a limit of 200 MFI) and SNR 1 (height of signal-to-noise ratios serve for differentiation of positive samples in disputed cases). Taking into account all monitored criteria, the most suitable condition for each parameter was chosen based on its achievement of the best ratings (see Supplementary Table S1) and further utilized within the optimized protocol.

## Results and Discussion

Different conditions were tested to evaluate whether deviations from the reference values (Fig. 2) had a significant impact on the MFI values and signal-to-noise ratios. If no impact was observed, no further conditions were tested. Optimized conditions for individual parameters were further used in the subsequent experiments.

**Figure 3:**
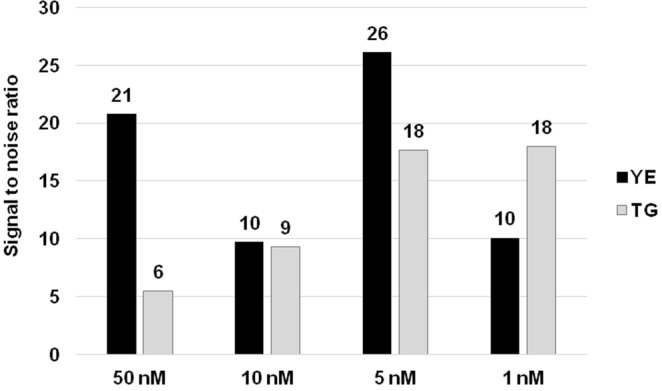
Determination of optimal concentration of MOLigo probes. The optimal concentration of each probe for both targets was found to be 5 nM. Other concentrations resulted in an increased background of NTCs, which led to a decrease in the signal-to-noise ratio. YE = *Y*. *enterocolitica;* TG = *T*. *gondii*.

**Figure 4:**
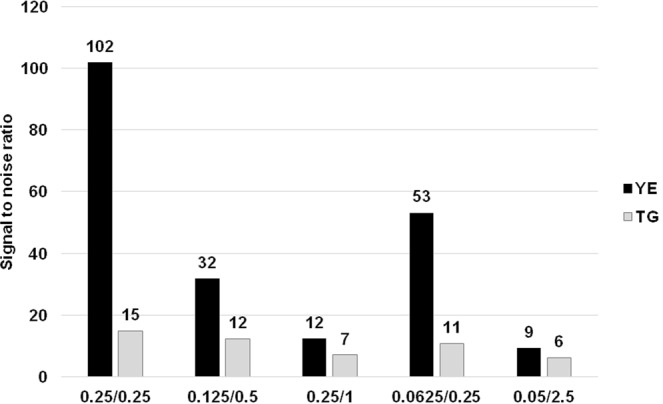
Optimization of the concentration of universal primers utilized in the singleplex PCR. The values on the x-axis represent FW primer/labeled REV primer concentration [μM]. The most suitable final concentration of FW primer was found to be 0.0625 μM and that of labeled REV primer 0.25 μM. YE = *Y*. *enterocolitica;* TG = *T*. *gondii*.

**Figure 5:**
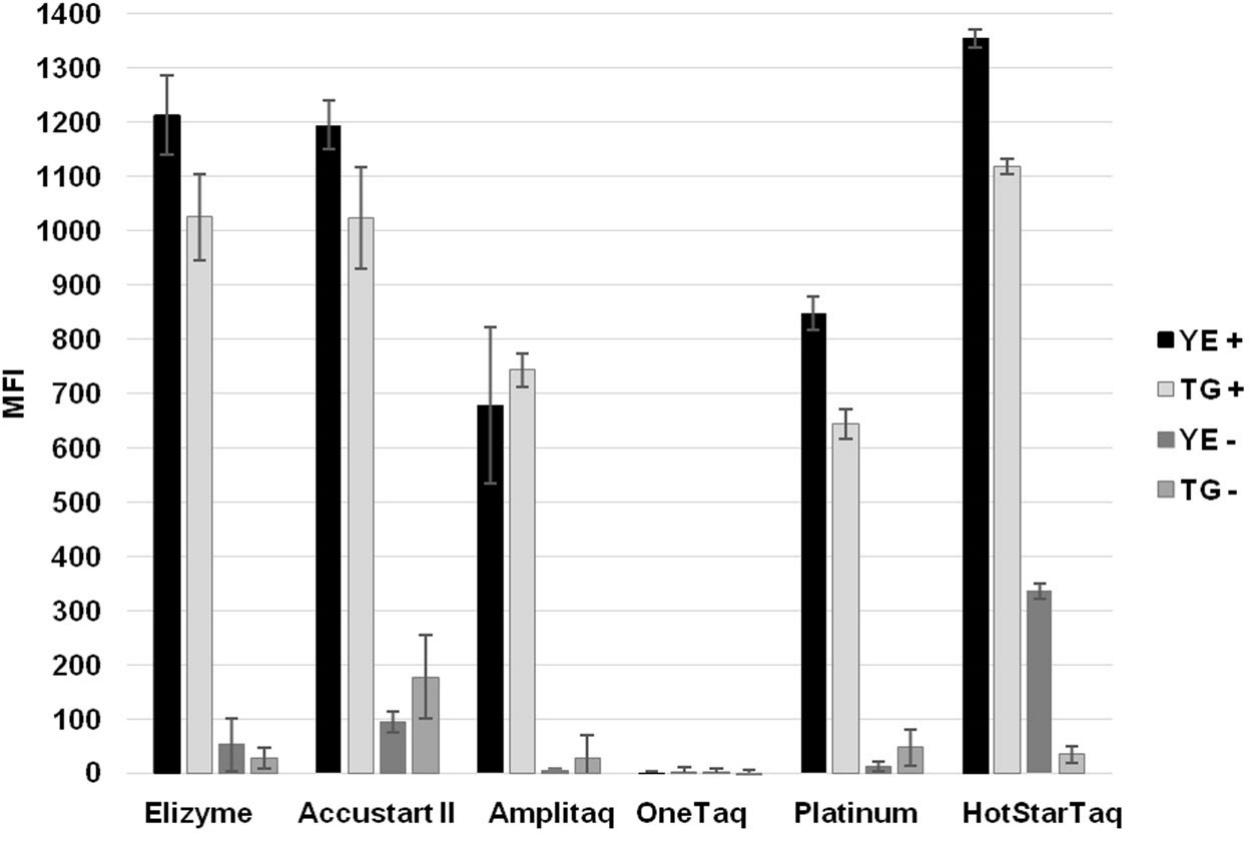
Impact evaluation of Master Mix used in singleplex PCR. In graph: Elizyme = 2X EliZyme HS Robust MIX (Elisabeth Pharmacon, Czech Republic); Accustart II = AccuStart II PCR ToughMix (QuantaBio, Massachusetts, USA); Amplitaq = AmpliTaq Gold 360 Master Mix (Thermo Fisher Scientific, Massachusetts, USA); OneTaq = OneTaq Hot Start 2X Master Mix with GC Buffer (New England BioLabs, Massachusetts, USA); Platinum = Platinum Hot Start PCR 2X Master Mix (Invitrogen, California, USA); HotStarTaq = HotStarTaq DNA Polymerase (Qiagen, Germany). YE = *Y*. *enterocolitica;* TG = *T*. *gondii*; plus sign in legend = positive sample; minus sign = NTC.

**Figure 6:**
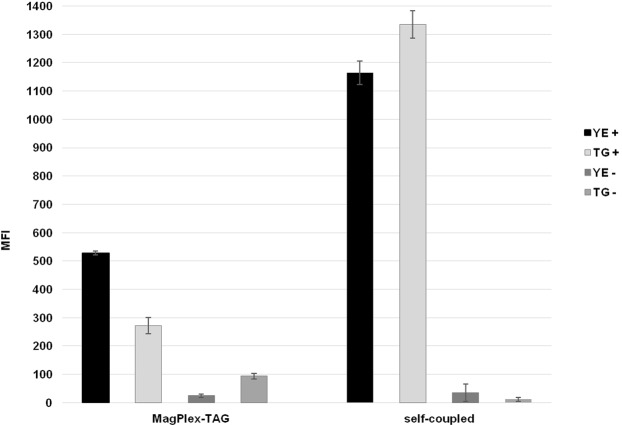
The efficiency of commercially pre-coupled MagPlex-TAG Microspheres in comparison to self-coupled MagPlex Microspheres. In *TG*, the obtained signal-to-noise ratio of 2.89 with the usage of pre-coupled microspheres did not fulfil the set criteria. YE = *Y*. *enterocolitica;* TG = *T*. *gondii*; plus sign in legend = positive sample; minus sign = NTC.

**Figure 7:**
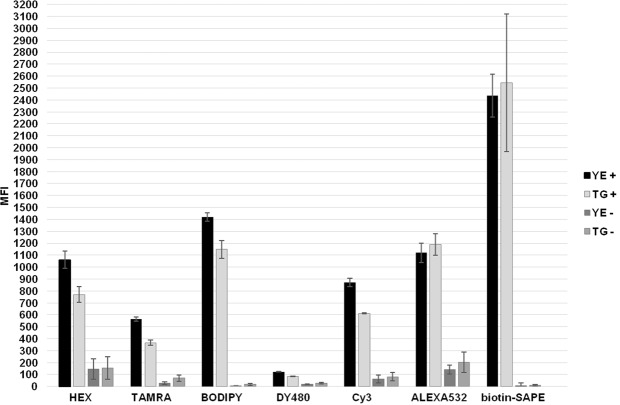
Comparison of efficiency of fluorescent reporters. 5′-end fluorophore modifications in graph: HEX = HEX (Eurofins Genomics, Luxembourg); TAMRA = TAMRA (Eurofins Genomics, Luxembourg); BODIPY = BODIPY-TMRX (Eurofins Genomics, Luxembourg); DY480 = DY480 (Eurofins Genomics, Luxembourg); Cy3 = Cy3 (Generi-Biotech, Czech Republic); ALEXA532 = Alexa Fluor532 (Invitrogen, California, USA); biotin-SAPE = biotin (Generi-Biotech, Czech Republic) and SAPE (Thermo Fisher Scientific, Massachusetts, USA). YE = *Y*. *enterocolitica;* TG = *T*. *gondii*; plus sign in legend = positive sample; minus sign = NTC.

**Figure 8:**
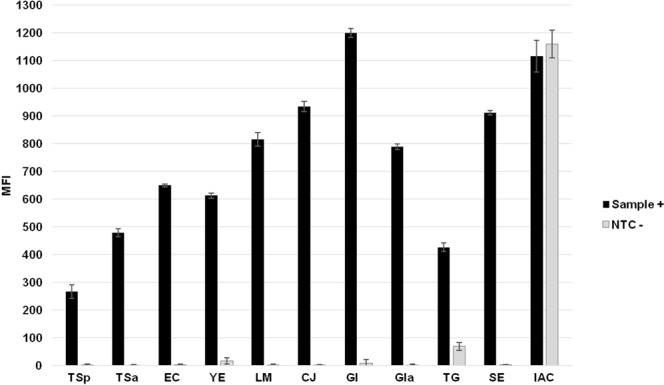
11-plex foodborne pathogens panel run with optimized protocol utilizing BODIPY-REV primer. In graph: TSp = *Trichinella spiralis;* TSa = *Taenia saginata*; EC = *Escherischia coli*; YE = *Y*. *enterocolitica;* LM = *Listeria monocytogenes;* CJ = *Campylobacter jejuni*; GI = *Giardia intestinalis* generic; GIa = *Giardia intestinalis* assemblage A; TG = *T*. *gondii*; SE = *Salmonella enterica;* IAC = internal amplification control.

In the case of bacteria (*YE*), a simple method for DNA isolation, such as heat lysis of a single colony, is preferable for routine purposes and was previously described for preparation of a DNA template for a MOL-PCR assay^6,7,10^. For eukaryotes (*TG*) and multicellular organisms, DNA isolation using extraction buffer was previously described and enables acquisition of very pure DNA^15,18^. In the present study, no differences were observed in the MOL-PCR optimization analyses when utilizing gDNA templates isolated by two distinct methods. The use of 2.5 μl of 10X diluted lysate and 2.5 ng of isolated DNA resulted in very high signals in comparison to NTCs (based on the results in Figs 3–8). In general, MOL-PCR is sensitive enough to allow usage of low concentrations of DNA (in the order of 1–0.1 ng of input material^6,10^); however, more concentrated DNA gives better results^7^ as there is a decrease of signal intensities with decreasing amounts of DNA^10^.

Song *et al*.^12^ developed the MOLigoDesigner web-based design tool (http://moligodesigner.lanl.gov) especially for the purposes of MOLigo probe design and probe quality check^6,10,12^; however, this helpful online tool is no longer available. Another possibility is the download of ThermoNucleotideBlast (http://public.lanl.gov/jgans/tntblast/)^4,13,19^, which is useful for melting temperature calculations or the purchase of Visual OMP software (DNA Software)^5,7^. Nevertheless, for an individual experienced with primer or probe design, common online tools such as Nucleotide BLAST and OligoAnalyzer 3.1 are sufficient. In some cases, it is complicated to find a suitable complementary sequence, which strictly conforms to all criteria, especially when the usable sequence region for the design of MOLigos is too short. The most problematic criterion during probe design appears to be the melting temperature. During probe design, it is recommended to design more variants of specific detection MOLigo pairs combining positive and negative (reverse complement) strands of the target complementary region as well as to switch TAG sequences between MOLigo1 and MOLigo2. Computational analysis indicated the absence of non-specific interactions; however, different results were obtained for each variant (see Supplementary Fig. S1 and Table S1). The utilization of a variant carrying the TAG sequence on MOLigo2 seems to be ideal, although no pattern in targeting a particular strand of target sequence was found. In initial testing, half of each assay product was visualized on an agarose gel stained with ethidium bromide and the second half hybridized to magnetic microspheres. Probe variants showing no ligation product or high NTC values were eliminated or redesigned.

### Optimization of the multiplex oligonucleotide ligation step

In the very first paper dealing with MOL-PCR by Deshpande *et al*.^4^, ligation and PCR were conducted in a single reaction using Ampligase (Epicentre, Wisconsin, USA) and Amplitaq Gold DNA polymerase (Roche, Switzerland). Polymerase was activated during the ligation step through a slow release mechanism and was able to subsequently perform the amplification of ligated MOLigos. However, follow-up studies by Thierry *et al*.^6^ and Stucki *et al*.^10^ were unsuccessful with such a workflow which resulted in high background noise. Therefore, the ligation step was performed separate from the PCR step optimization experiments and high signals compared to the background noise were obtained after optimization.

The ligation step which constitutes the actual detection event includes a specific MOLigo pair intended for the detection of a particular marker. The total concentration of probes in a MOL-PCR reaction seems to be a crucial factor. In the literature, the concentration ranges from 1 nM^7^ up to 10 μM^6^ (Fig. 2). For evaluation of the probe concentration effect on the signal-to-noise ratio, the concentration of each probe in the ligation mix was tested at 1 nM, 5 nM^4^, 10 nM, 50 nM, and 1 and 10 μM. Based on numerical data evaluation (see Supplementary Table S1), the optimal concentration of MOLigo probes was established at the very low level of 5 nM of each probe as other concentrations resulted in increased MFI values of NTCs and thus decreases in signal-to-noise ratios (Fig. 3). If higher concentrations of 1 or 10 μM were used, no ligation product was detected on an agarose gel. Low concentrations are important especially in assays with high levels of multiplexing, e.g. the same concentration of 5 nM was used in a biothreat panel combining more than 10-plex assays^4^, while an even lower concentration of 2 nM was utilized in an 8-plex assay for SNP-typing (three probes per SNP) of the *Mycobacterium tuberculosis* Complex^10^ and in three MOL-PCR assays for subtyping of *Salmonella* Typhimurium by 52 molecular markers^5^.

The ligation temperature should be considered in order to ensure high specificity and efficiency of the detection event, since the usage of higher temperatures (near the melting temperatures of the used MOLigos) increases the annealing stringency^20^. To determine the temperature that most efficiently ensured the formation of a covalent bond between the two MOLigos after hybridization to the target sequence, and its impact on the signal-to-noise ratio, a temperature gradient from 55 to 70 °C was tested. While maximum MFIs varied only very little, temperature had an impact on NTCs; based on numerical data evaluation (see Supplementary Table S1) ligation temperature between 59–60 °C was considered as the most suitable. This value is higher than in the majority of previously reported data, where a ligation temperature of only 50 °C was used in a 10-plex biothreat panel^4^ and an 8-plex assay for *M*. *tuberculosis*^10^. This might reflect different requirements for optimal performance of each thermostable ligation enzyme; in the case of Hifi *Taq* DNA ligase, typical nick-ligation procedures should be performed at 60 °C. Therefore, a temperature range of about 59–60 °C fulfills the prerequisites for the best activity and fidelity of the enzyme based on oligo-template properties and ensures the reduction of errors caused by mismatched base pairs.

According to the manufacturers’ recommendation, a novel thermostable Hifi *Taq* DNA ligase may efficiently seal nicks in DNA when incubated at the optimal ligation temperature for 15 minutes or for repeat cycles of the denaturation and annealing steps. In an attempt to shorten the working protocol, the longer cycling protocol including initial denaturation for 10 min at 95 °C followed by 20 cycles of 30 s at 95 °C and 1 min at 60 °C was compared with a shorter incubation protocol consisting of initial denaturation for 5 min at 95 °C followed by 15 min incubation at 60 °C. This comparison showed (see Supplementary Fig. S2) that linear amplification occurring during cycling does not have any significant effect on the fluorescent intensity of positive samples; however, such amplification suppresses non-specificity in NTCs and thus increases signal-to-noise ratios. Based on numerical data evaluation (see Supplementary Table S1), the ligation protocol based on cycling was further utilized.

The best efficiency and lowest background noise was obtained with the Hifi *Taq* DNA Ligase (see Supplementary Table S1), of which it has been claimed that it is the NAD^+^-dependent DNA ligase with the highest fidelity currently available commercially^21^. There is the possibility to use Ampligase Thermostable DNA Ligase (Epicentre) or *Taq* DNA Ligase (New England BioLabs, Massachusetts, USA) instead, although the fidelity is greatly reduced^21^. Also, the usage of Ampligase or *Taq* ligase may result in the reduced effectiveness of some previously functioning MOLigo pairs (in the case of *YE;* see Supplementary Fig. S3) caused by a significant increase in NTCs; such pairs would thus become unusable and would have to be redesigned.

To ensure optimal performance of the ligation reaction and to suppress cross-reactivity in problematic MOLigo pairs, fish sperm DNA can be added to the ligation reaction mix. The addition of 50 ng of DNA from fish sperm (SERVA, Germany) in 1X TE buffer markedly reduced NTCs in a problematic detection system (see Supplementary Fig. S4) and increased the signal-to-noise ratio from less than 4 to up to 39, thus allowing data interpretation and further use of such a system in future assays. The exact mechanism of action is unknown, but one can assume that carrier DNA prevents the non-specific binding of MOLigos to other DNA sequences or attachment to tube surfaces^22^, which is a common reason for carrier DNA addition to mastermixes. Utlization of carrier DNA in MOL-PCR was previously described by Woods *et al*.^13^, who included carrier DNA at a concentration of 0.15 mg/ml in the ligation mix of their 11-plex assay for Shiga toxin-producing *E*. *coli*. The positive properties of carrier DNA have also been described with respect to DNA isolation, where the addition increased the yield of DNA^23^.

### Optimization of singleplex PCR

Subsequent singleplex PCR amplification was performed using the successfully ligated MOLigos as a template and the universal primer pair, with the REV primer carrying a fluorescent reporter. Most ligase reaction buffers usually contain white precipitate particles, which need to be fully dissolved by warming in hand and/or vigorous shaking. Buffers should not also be subject to repeated freeze-thawing, which may distort results leading to high MFI values of NTCs. These particles are likely to consist of dithiothreitol (DTT) used to preserve enzyme stability during *in vitro* reactions. DTT was recently described as a possible cause of markedly increased background, and thereby reduced signal-to-noise ratios^24^.

The significant drop in signal when incorporating higher loads of PCR products in the microsphere hybridization reaction was described previously^6^. To limit possible bias during the hybridization of labeled MOL-PCR product to microspheres, the concentration of universal primers was altered with the aim of producing predominantly fluorescently-labeled single-stranded products. Universal primers (Table 1) were adopted from Thierry *et al*.^13^ and four-fold less FW primer than BODIPY-TMRX labeled REV primer at concentrations of 0.125/0.5 μM, 0.25/1 μM, 0.0625/0.25 μM, and 0.05/2.5 μM were used in comparison to a uniform concentration of both primers of 0.25 μM. This asymmetric PCR amplification strategy improved hybridization efficiency and increased maximal MFI values for positive samples (see Supplementary Fig. S5). Fifty-fold less^6^ FW primer (0.05/2.5 μM) led to the highest MFI values; however, a noticeable increase of MFI values of NTCs was also observed. Based on numerical data evaluation (see Supplementary Table S1), final concentrations of 0.0625 μM for FW primer and 0.25 μM for labeled REV primer were considered as the most efficient despite the fact that the uniform concentration of 0.25 μM for both primers resulted in higher signal-to-noise ratios (Fig. 4) but considerably lower MFI values.

Another important factor influencing the effectiveness and cost of the MOL-PCR reaction is the choice of the master mix. In order to find a balance, the performance of six different master mixes was tested (Fig. 5), including the previously utilized HotStarTaq DNA Polymerase^5–7^ and the upgraded AmpliTaq Gold 360 Master Mix^4,13^. The MOL-PCR reaction using OneTaq HotStart was not successful and no products were detected, since the results did not meet even one of the interpretative criteria. HotStarTaq DNA Polymerase, on the other hand, reached the highest MFI values. However, the increase of NTC noise was significant in *YE* and, with a signal-to-noise ratio of 4, was on the border of positivity. Based on numerical data evaluation (see Supplementary Table S1), the highest efficacy was achieved with the EliZyme HS Robust mix, which in addition, offers time savings through the shorter cycling profile and compound complexity of stock solution.

### Optimization of hybridization step and MAGPIX analysis

In the last step, the specific hybridization of fluorescently labeled MOL-PCR product to the magnetic microspheres enables detection in a MAGPIX reader. This final step requires several parameters to be considered such as the type and number of magnetic microspheres, choice of fluorescent reporter or working protocol. As presented in Fig. 2, all previously developed MOL-PCR assays utilized commercially available pre-coupled MagPlex-TAG Microspheres or MicroPlex Microspheres (Luminex Corp., Texas, USA). However, the fluorescent intensity of pre-coupled microspheres (Fig. 6) was less than half that achieved by self-coupled MagPlex microspheres used in other experiments and, with a signal-to-noise ratio of less than 3 in the case of *TG*, did not fulfil the stipulated criteria. Based on numerical data evaluation (see Supplementary Table S1), self-coupled MagPlex Microspheres were considered as more efficient and for this reason were utilized within all optimization experiments.

Findings concerning microsphere number per reaction in ligation assays are based on analyses on flow cytometer devices. The general recommendation for obtaining accurate results is to use up to 2,500 input microspheres^25^ per reaction and to count 100 events^26^. Bruse *et al*.^27^ concluded that an input of 200 microspheres and a minimum of 20 events provide the same results as higher amounts that have been recommended^27^ and Jacobson *et al*.^28^ went even further and declared that as few as 10 microspheres are sufficient under appropriate conditions^28^. However, flow cytometer devices differ from MAGPIX readers in their mechanisms of measurement and little data are available for this basic instrument. Woods *et al*.^13^ screened Shiga toxin-producing *E*. *coli* using 1,000 microspheres per reaction and counted a minimum of 100 events in their 11-plex assay. Wuyts *et al*.^5,7^ used 750 input microspheres for subtyping of *Salmonella* Typhimurium and counted a minimum of 50 events. Before setting this parameter, they tested 375, 750, and 2,500 microspheres per reaction and obtained the best results with 750 microspheres, since the lower number resulted in counts of below 50. For deeper exploration, we tested 350, 750, 1,000, 1,500, 2,500, 3,500, and 5,000^4^ microspheres per reaction with a minimum count set to 50 events (see Supplementary Fig. S6). Regarding high MFI, low NTCs (high signal-to-noise ratios), and minimal deviations between measurements, 2,500 microspheres per reaction showed slightly better efficiency. Moreover, lower numbers resulted in counts of less than 50 (see Supplementary Table S2); specifically, 350 microspheres were on average below the minimum event count in 97% of samples, 750 microspheres in 64%, 1,000 microspheres in 76%, and 1,500 in 38% of samples. Meanwhile, the use of 2,500 and more microspheres resulted in event counts that were below the minimum in less than 3% of samples. Based on numerical data evaluation (see Supplementary Table S3), where also the criterion of microsphere count below 50 was included, we conclude that the reference number of 2,500 microspheres per reaction generates the most reliable data.

The choice of fluorescent reporter dye may also significantly affect the resulting data. SAPE is recommended by Luminex as the most efficient reporter (100% relative fluorescence intensity) for read-outs on a MAGPIX^29^. The “Relative reporter intensities”^29^ graph shows that other relevant reporters lag far behind, with Alexa Fluor532 (28%) coming a distant second, Cy3 (19%) third and BODIPY-TMRX (7%) determined to be the sixth most suitable dye. We re-evaluated this finding and tested other available dyes in an experiment including universal REV primers labeled at the 5′-end with seven different molecules whose spectra are relevant to MAGPIX diodes: HEX, TAMRA, BODIPY-TMRX, DY480, Cy3, Alexa Fluor532, and biotin binding with SAPE (Fig. 7). The highest MFI values and signal-to-noise ratios (see Supplementary Fig. S7) were obtained with biotin-SAPE usage as previously stated. However, SAPE utilization also showed the greatest deviations between measurements reaching a difference in MFI of up to 1,000. Based on numerical data evaluation (see Supplementary Table S1) the performance of Alexa Fluor532 and Cy3, previously reported as another suitable dyes for MOL-PCR assay^4,10^, were greatly overcome by BODIPY-TMRX. On the other hand, DY480 did not meet either the minimum MFI of at least 200 or a signal-to-noise ratio of at least 4 and was evaluated as inappropriate for analysis on a MAGPIX reader. It is worth noting that the use of a direct label on a universal REV primer holds an advantage over indirect biotin-SAPE labeling in that only one labeled primer that marks all successfully ligated probes during a PCR reaction is needed; with biotinylation usage, meanwhile, a further incubation step of SAPE binding is required. This provision ensures time savings, reduces the financial burden, and diminishes the risk of cross contamination. For this reason and also in order to minimize deviations in measurements, we have found BODIPY-TMRX to be the most suitable and efficient fluorescent reporter for our MOL-PCR assay, and we utilized this dye for all optimization experiments presented within the study.

An alternative strategy aimed at increasing the fluorescence intensity based on incorporation of labeled nucleotides into target DNA sequence during the PCR step, was tested using biotin-16-dCTPs. Biotin-dNTPs are commonly used for allele-specific primer extension assays^30,31^ on MAGPIX and its potential for the MOL-PCR assay was tested. The results showed significantly increased MFI values reaching tens of thousands in positive samples (see Supplementary Fig. S8). However, with NTCs of about 1,000 MFI, the signal-to-noise ratios were below the efficiency of SAPE (biotinylated REV primer) or BODIPY and with the regard to the higher price of biotinylated nucleotides and requirement for a subsequent incubation step, were not rated as the most appropriate labeling variant for MOL-PCR products. To conclude, fluorescent reporters other than SAPE showed lower absolute fluorescence; nevertheless, the performance is stable during the course of measurements, interpretative criteria are clear, and the working protocol is short.

### Optimized conditions in the multiplex assay

To demonstrate the efficiency of the parameters optimized in the singleplex MOL-PCR systems and to prove the functionality of the described working protocol in a multiplex format, a model 11-plex FBP panel was created (Fig. 8). It is important to mention that this multiplex assay does not have a practical meaning as the sample with such pathogen composition is unlikely to be found and systems will be reorganized for future use.

IAC proved itself to be a reliable indicator of the accuracy of the results and all bacterial and parasitic systems fulfilled the set criteria (see Supplementary Fig. S9), proving their functionality for future experiments. Nevertheless, some systems showed a sharp decline in signal-to-noise ratios manifested by both a decrease of MFI values in positive samples and an increase of NTCs in comparison to their performance in singleplex assays, e.g. *TG* had a ratio of 6 in multiplex but reached a ratio of 69 in a singleplex test with fluorescent dyes (see Supplementary Figs S7 and S9); for similar issues might be a solution a division of the multiplex panel into separate groups, where individual systems would be combined on the basis of species relatedness or possibility of their common occurrence in particular matrix^32^, where the systems included would be tested for mutual interactions. At higher levels of multiplexing (more than 20 targets), the limit of detection (0.1 ng of input DNA as described above) might be reduced for individual targets when compared to their values in singleplex^33^.

Within this manuscript we do not include the limits of detection or specificity tests for particular systems of our model multiplex assay, these evaluations must be performed after optimization of MOL-PCR and MAGPIX analysis as whole, before implementation of the specific multiplex panel into routine practice.

### Accessibility criteria

Microsphere-based assays based on xMAP technology (Luminex Corp., Texas, USA) require the purchase of a Luminex instrument. The most basic and also cheapest instrumentation suitable for such assays is a MAGPIX reader utilizing magnetic microspheres. According to the analyte of interest the MAGPIX is adjustable for a wide range of applications, e.g. nucleic acid assays and protein or immuno-assays. Moreover, many laboratories already have access to such instrumentation.

The most significant expenses of a typical Luminex-based assay are the ligation enzyme, PCR master mix, and microspheres. Regarding the MAGPIX reader, MagPlex-TAG Microspheres (2.5 × 10^6^ microspheres/ml) are commercially available. With a 1 ml volume of microsphere stock solution and when using a microsphere mix with 2,500 microspheres/reaction, these commercial microspheres are sufficient for 1,000 reactions. In comparison, self-coupled MagPlex Microspheres are sufficient for at least 4,000 reactions; the necessary number of microspheres can be prepared in a single coupling procedure. Even with the inclusion of costs for reagents required for coating and labor, the self-coupled beads are still financially preferable (4% of the cost per reaction) over commercial microspheres.

Using the optimized MOL-PCR method described in the present study, the cost per reaction for detection of one marker is approximately €2. The costs drop even lower when multiplexing. The highest fidelity was proven when using Hifi *Taq* DNA Ligase, which represents the state-of-the-art in the field of thermostable ligases; however, this novel ligase is a factor that significantly increases the cost of analysis. If the price is the significant criterion for laboratory purposes, Ampligase or *Taq* DNA Ligase might be used as an alternative. Nevertheless, the reduced efficiency described above has to be taken into account. When using Ampligase, the price for one sample reaches €0.68, which roughly corresponds to €0.8 per assay, and similar to what was calculated by Stucki *et al*.^10^. However, the price of maintenance reagents necessary for proper operation of the MAGPIX reader should be also taken into account. It is in particular a Calibration kit, which is used approximately once a week and a Verification kit, which has to be used after each instrument startup; therefore an amount of approximately 20 cents per sample (considering a 96-well plate) should be included.

Another important factor is the time needed to perform the assay. In comparison to the closely related MLPA, which requires about 20 hours to complete, the MOL-PCR assay can be performed very rapidly^4^. MLPA remains, even in the version adapted to the microsphere array and when using the same detection platform as MOL-PCR, tedious due to the number of steps or necessity for overnight hybridization^34^. The MOL-PCR assay is designed to be performed in 96-well plates, while simultaneously allowing each well to be screened for up to 50 markers, and provides high-confidence results within 6 h^6,12^. Hands-on-time might be further shortened to less than 4 hours by usage of directly labeled universal primers and with the assay workflow described above (see overview in Fig. 2).

## Conclusion

Modern diagnostics should meet current demands with respect to accuracy, rapidity, and complexity, in order to combine all necessary data within a simple methodology. Although the multiplexing of PCR diagnostic assays is desirable, there are technological limits to multiplexing in various derivatives of PCR. MOL-PCR adapted to Luminex instruments has the potential to overcome these limits and has proven to be suitable for a number of diagnostic approaches relying on a complex and multiplex analysis of the sample. Further, there is no necessity to change technology as the PCR cycler is nowadays present in each laboratory and the technique can be adapted to current instrumentation. However, even MOL-PCRs whose suitability has been proven in previous studies must be precisely optimized in order to serve as a robust and functional tool in routine diagnostics. To the best of our knowledge there is no complex study available which would comprehensibly identify all parameters needed to design a reliable in-house multiplex MOL-PCR assay and which would characterize the impact of each parameter on the performance of the assay. Through the present study as a guideline to the optimization process, we, therefore, intend to bring this approach closer to the wider body of professionals and to facilitate the spread of the MOL-PCR technology to complex routine microbial analysis of food.

## Supplementary information

Supplementary Information

## Data Availability

All data generated or analyzed during this study are included in this published article (and its Supplementary Information file).
