# Author Correction: A novel perspective on MOL-PCR optimization and MAGPIX analysis of in-house multiplex foodborne pathogens detection assay

**DOI:** 10.1038/s41598-019-42194-x

**Published:** 2019-04-04

**Authors:** Nikol Reslova, Veronika Huvarova, Jakub Hrdy, Martin Kasny, petr Kralik

**Affiliations:** 1 Veterinary Research Institute, Department of Food and Feed Safety, Hudcova 296/70, 621 00 Brno, Czech Republic; 2 Faculty of Science, Department of Botany and Zoology, Masaryk University, Kotlářská 2, 611 37 Brno, Czech Republic; 3 Faculty of Science, Department of Experimental Biology, Masaryk University, Kamenice 753/5, 625 00 Brno, Czech Republic

Correction to: *Scientific Reports* 10.1038/s41598-019-40035-5, published online 25 February 2019

This Article contains typographical errors in the Acknowledgements section.

“The work was supported by the Ministry of Education, Youth and Sports of the Czech Republic (LO1218), by the Ministry of Agriculture (RO0516), Ministry of the Interior of the Czech Republic (VI20152020044), and by Masaryk University institutional support (MUNI/A/0816/2017).”

should read:

“The work was supported by the Ministry of Education, Youth and Sports of the Czech Republic (LO1218), by the Ministry of Agriculture (RO0518), Ministry of the Interior of the Czech Republic (VI20152020044), and by Masaryk University institutional support (MUNI/A/0816/2017).”

