# Supplementary material for: A novel perspective on MOL-PCR optimization and MAGPIX analysis of in-house multiplex foodborne pathogens detection assay
Source: Sci Rep. 2019 Feb 25;9:2719. doi: 10.1038/s41598-019-40035-5 (PMC6389906; doi:10.1038/s41598-019-40035-5)

A novel perspective on MOL-PCR optimization and MAGPIX analysis of in-house multiplex foodborne pathogens detection assay

Nikol Reslová, Veronika Huvarová, Jakub Hrdý, Martin Kašný, Petr Králík

Table S1: Numerical evaluation of individual tested conditions for both targets *YE* and *TG*. MFI of the positive samples (MFI+), MFI of the NTCs (MFI-) and height of the signal-to-noise ratio (SNR). Weight of each criterion: MFI+ 3, MFI- 2 and SNR 1. The lowest weighted mean represents the most sufficient condition (highlighted in yellow), which was further utilized within the optimized protocol.

Condition	*YE* MFI +	*YE* MFI -	*TG* MFI +	*TG* MFI -	*YE* SNR	*TG* SNR	Weighted mean
**MOLigo probe variants (Figure S1)**							
**MOL 2/-**	1	1	-	-	1	-	**1.00**
**MOL 2/+**	4	2	-	-	3	-	**3.17**
**MOL 1/-**	2	4	-	-	3	-	**2.83**
**MOL 1/+**	3	3	-	-	3	-	**3.00**
**MOLigo probe concentration (Figure 2)**							
**50 nM**	1	2	1	4	2	4	**2.00**
**10 nM**	2.5	3.5	2.5	3	3.5	3	**2.88**
**5 nM**	2.5	1	2.5	1.5	1	1.5	**1.88**
**1 nM**	2.5	3.5	4	1.5	3.5	1.5	**2.88**
**Ligation temperature**							
**55°C**	4	6	6	4.5	7	4	**5.17**
**56.3°C**	4	9	1.5	7	9	7	**5.38**
**59.2°C**	1.5	6	3.5	6	5	5	**4.08**
**59.8°C**	10	2	8.5	1.5	2	1	**5.46**
**61.2°C**	7.5	4	10	3	4	3	**6.13**
**62°C**	9	3	7	1.5	3	2	**5.17**
**63.3°C**	7.5	1	8.5	4.5	1	6	**5.50**
**63.9°C**	4	6	3.5	9	6	9	**5.63**
**67.7°C**	6	8	1.5	8	8	8	**5.88**
**69.7°C**	1.5	10	5	10	10	10	**6.63**
**Cycling versus incubation of the ligation reaction (Figure S2)**							
**20 cycles**	1	1	2	1	1	1	**1.25**
**15 min incubation**	2	2	1	2	2	2	**1.75**
**DNA ligases (Figure S3)**							
**Hifi *Taq* ligase**	1	1	1	3	1	2	**1.42**

***Taq* ligase**	2	3	3	2	2.5	3	**2.54**
**Ampligase**	3	2	2	1	2.5	1	**2.04**
**Concentration of universal primers (Figure 3 and Figure S5)**							
**0.25/0.25 µM**	5	1	5	1	1	1	**3.00**
**0.125/0.5 µM**	3.5	3	3.5	3	3	2	**3.17**
**0.25/1 µM**	2	4	2	4	4	4	**3.00**
**0.0625/0.25 µM**	3.5	2	3.5	2	2	3	**2.83**
**0.05/2.5 µM**	1	5	1	5	5	5	**3.00**
**PCR master mixes (Figure 4)**							
**Elizyme**	2.5	4	2.5	2.5	3	1	**2.67**
**Accustart II PCR TOughMix**	2.5	5	2.5	6	4	5	**3.83**
**Amplitaq Gold**	5	2	4	2.5	1	3	**3.33**
**OneTaq HotStart**	6	1	6	1	6	6	**4.33**
**Platinum HotStart**	4	3	5	5	2	4	**4.08**
**HotStarTaq**	1	6	1	4	5	2	**2.75**
**Pre-coupled versus self-coupled microspheres (Figure 5)**							
**MagPlex-TAG**	2	1	2	2	2	2	**1.83**
**Self-coupled**	1	2	1	1	1	1	**1.17**
**Fluorescent reporter dyes (Figure 6 and Figure S7)**							
**HEX**	4	6	4	6	6	5.5	**5.0**
**TAMRA**	6	4	6	4	3	5.5	**5.0**
**BODIPY-TMRX**	2	1	3	2	2	2	**2.1**
**DY480**	7	3	7	3	7	7	**5.7**
**Cy3**	5	5	5	5	4	3	**4.8**
**ALEXA532**	3	7	2	7	5	4	**4.3**
**biotin-SAPE**	1	2	1	1	1	1	**1.2**

Figure S1: Performance of different variants of specific MOLigo detection pairs using biotin-SAPE. MOL2/- = TAG on MOLigo2, targeted to the negative strand; MOL2/+ = TAG on MOLigo2, targeted to the positive strand; MOL1/- = TAG on MOLigo1, targeted to the negative strand; MOL1/+ **=** TAG on MOLigo1, targeted to the positive strand; YE = *Y. enterocolitica;* plus sign in legend = positive sample; minus sign = NTC.

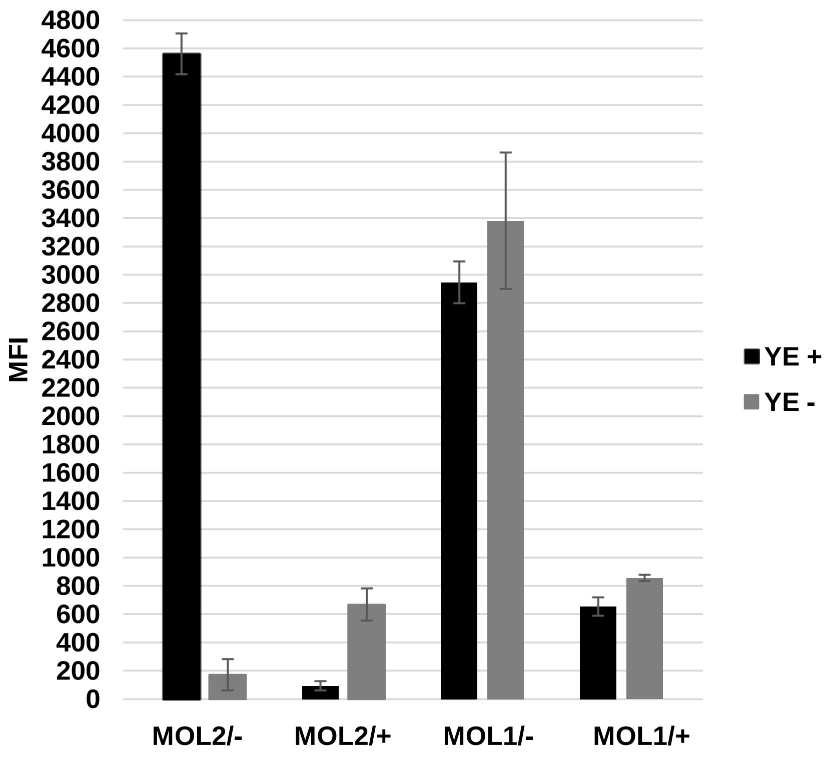

Figure S2: Comparison of the effect of cycling versus incubation of the ligation reaction. Twenty cycles of denaturation/annealing were compared to 15 minutes of incubation using the optimized ligation temperature of 60°C . From the values depicted in the graph, signal-to-noise ratios reaching 10 in YE and 15 in TG in the case of cycling and 8 in YE and 5 in TG in the case of incubation were calculated. YE = *Y. enterocolitica;* TG = *T. gondii*; plus sign in legend = positive sample; minus sign = NTC.

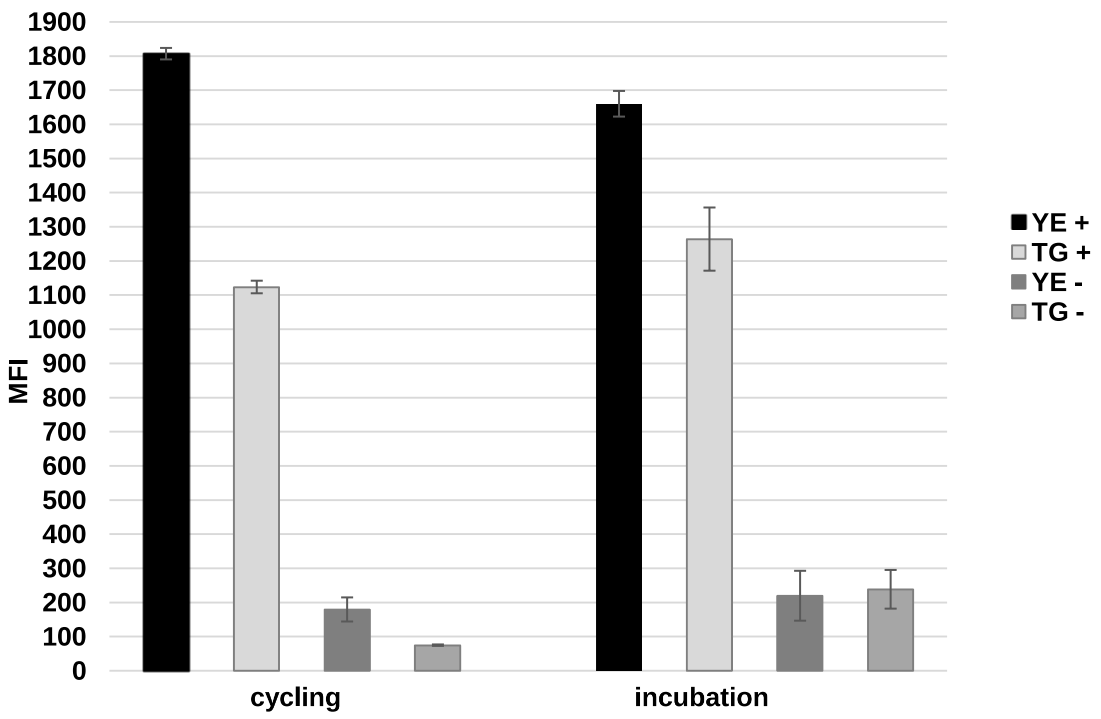

Figure S3: Comparison of thermostable ligases available for utilization in MOL-PCR assays. Abbreviated names in the graph correspond to Hifi *Taq* DNA Ligase (New England BioLabs, Massachusetts, USA); *Taq* DNA Ligase (New England BioLabs, Massachusetts, USA) and Ampligase Thermostable DNA Ligase (Epicentre). From the values depicted in the graph signal-to-noise ratios reaching 66 in YE and 11 in TG (Hifi *Taq* ligase); 1 in YE and 8 in TG (*Taq* ligase); 2 in YE and 19 in TG (Ampligase) were calculated. YE = *Y. enterocolitica;* TG = *T. gondii*; plus sign in legend = positive sample; minus sign = NTC.

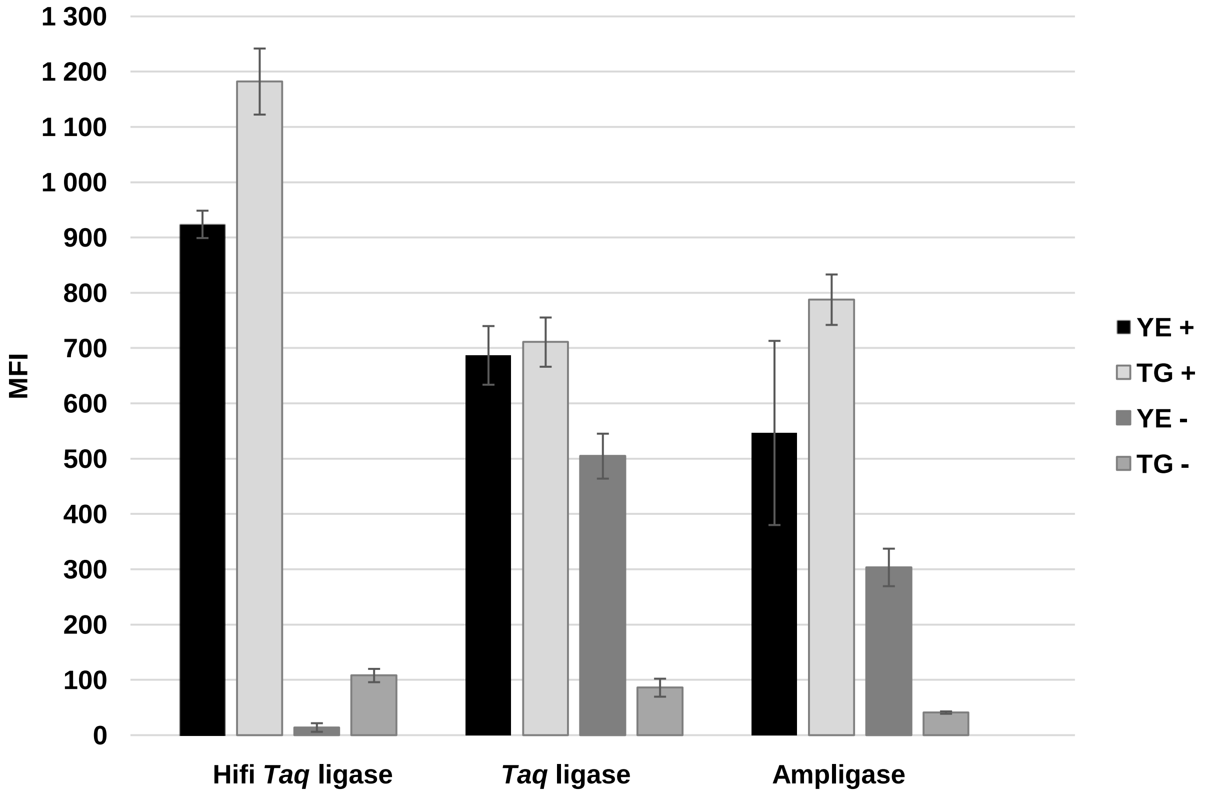

Figure S4: The influence of addition of fish sperm carrier DNA to the ligation reaction mix. From the values depicted in the graph a signal-to-noise ratio of 3.7 when using only water and a ratio of 39 with usage of fish sperm were calculated. TSp =*Trichinella spiralis;* plus sign in legend = positive sample; minus sign = NTC.

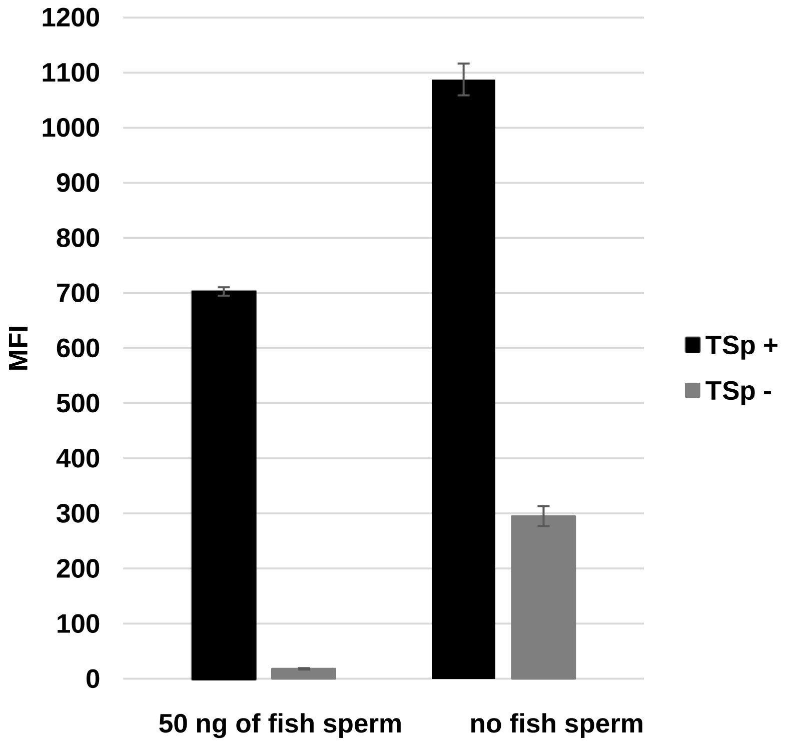

Figure S5: Asymmetric PCR amplification strategy using altered concentrations of universal primers. YE = *Y. enterocolitica;* TG = *T. gondii*.

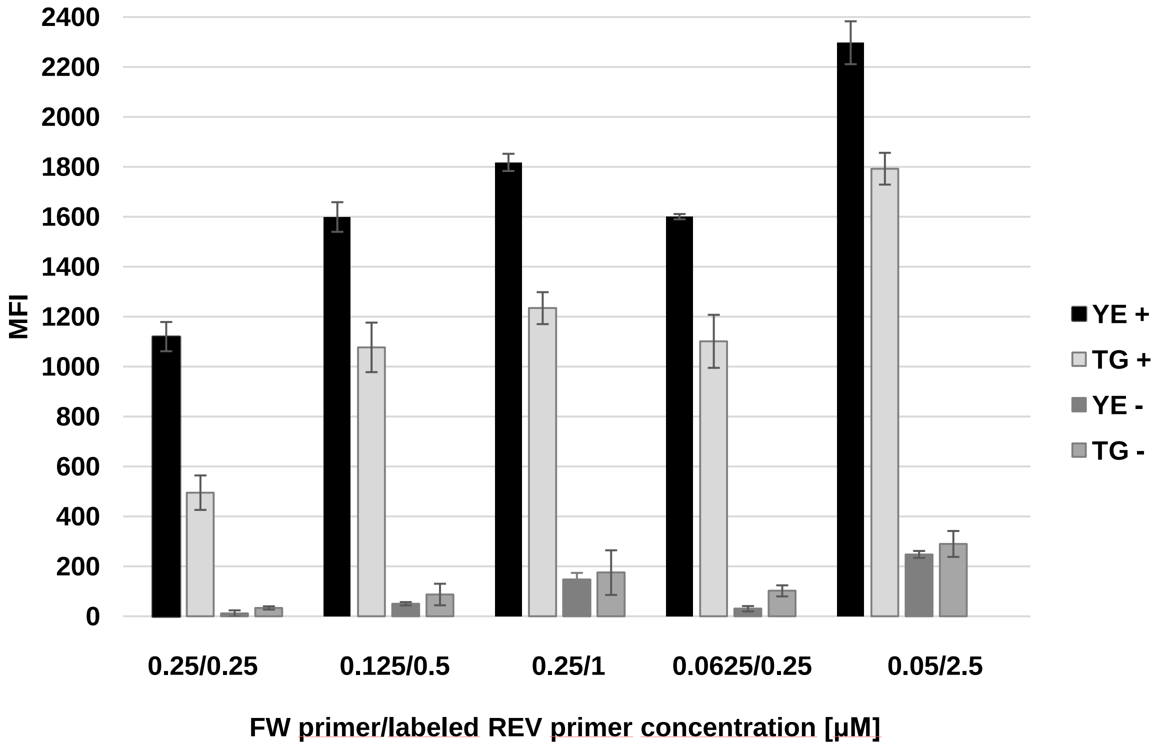

Figure S6: Consideration of microsphere number per reaction.

A) **MFI values of positive samples and NTCs using different numbers of microspheres per reaction.** YE = *Y. enterocolitica;* TG = *T. gondii*; plus sign in legend = positive sample; minus sign = NTC.

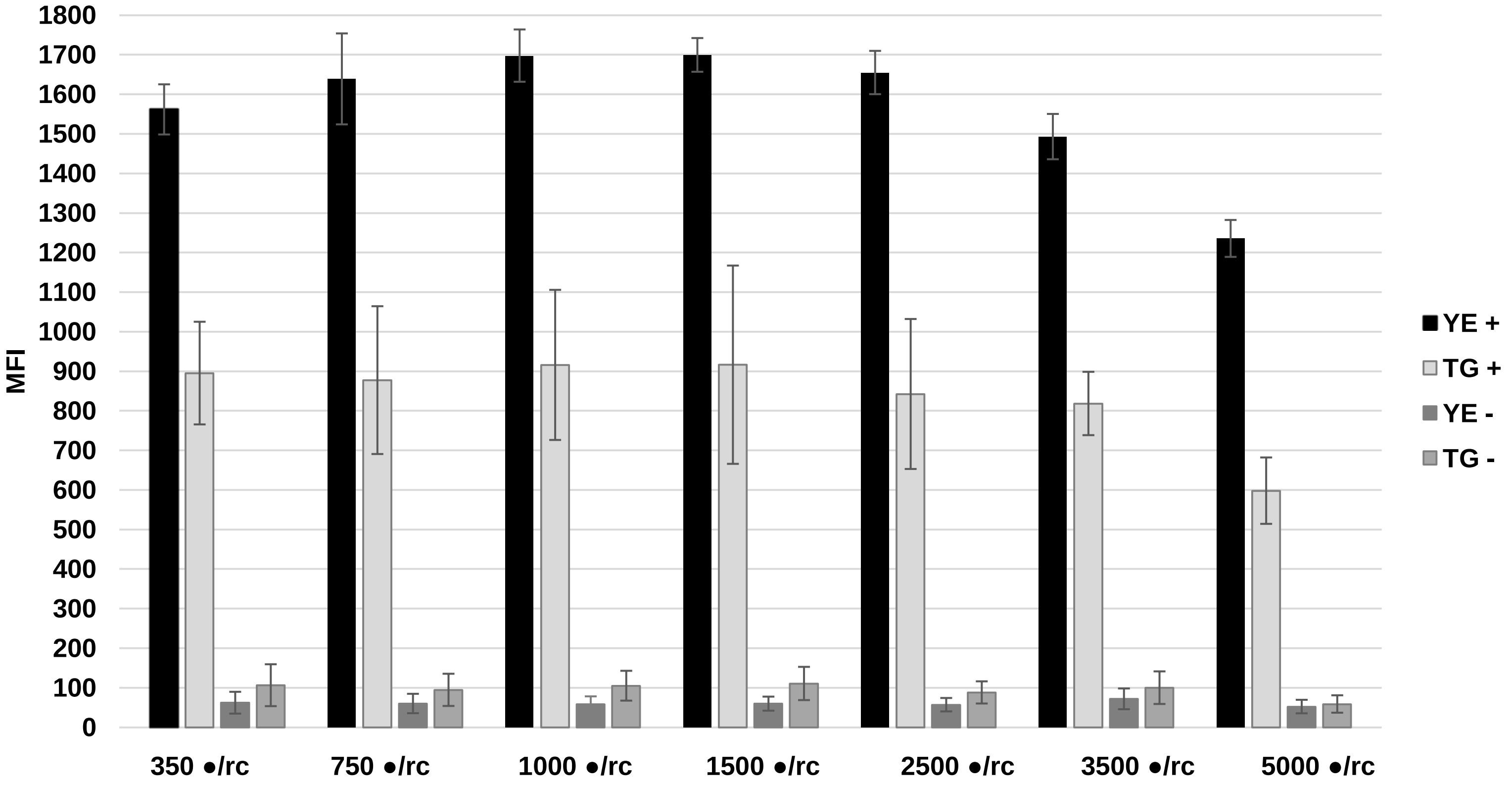

B) Signal-to-noise ratios with usage of different numbers of microspheres per reaction. YE = *Y. enterocolitica;* TG = *T. gondii*.

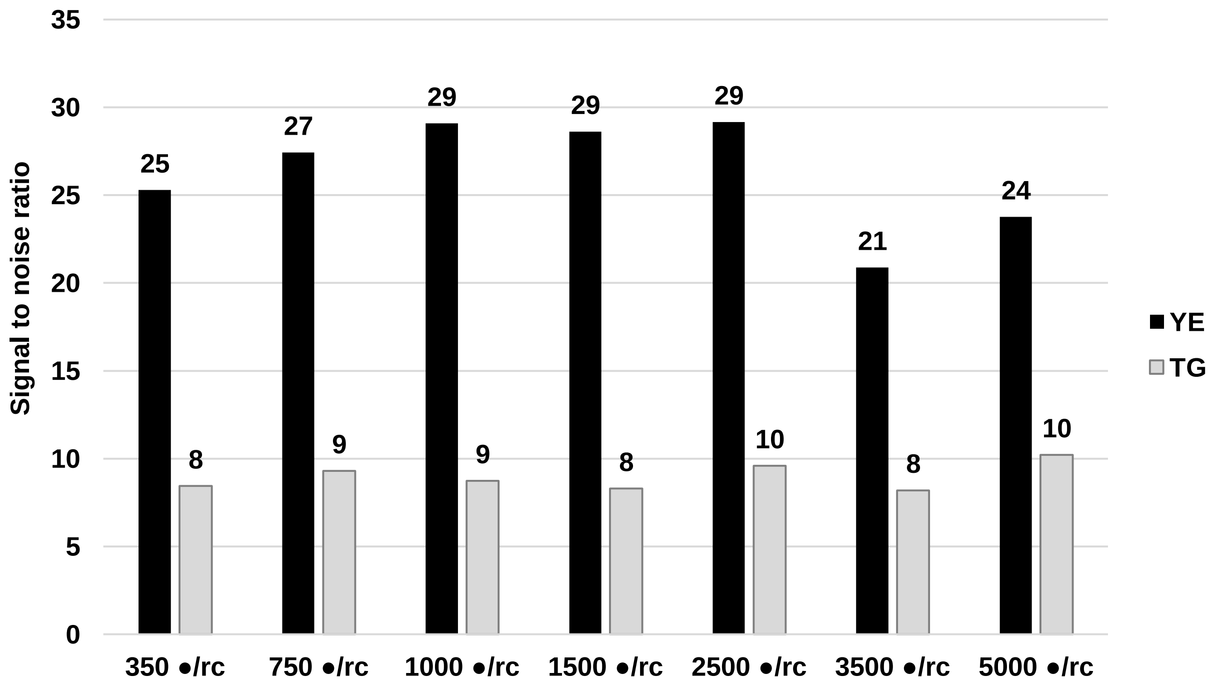

Table S2: Table showing average microsphere number per reaction detected during analysis and the respective percentages of samples below the set count of 50. In all samples at least one microsphere was detected. * = only one of the quadruplicates.

**Microsphere set**	**Microsphere input per reaction** [microsphere count; below set count 50]						
	**350**	**750**	**1,000**	**1,500**	**2,500**	**3,500**	**5,000**
**region 19**	23; 94%	63; 28%	48; 63%	80; 19%	240; 0%	176; 0%	581; 0%
**region 34**	6; 100%	21; 100%	24; 88%	41; 56%	121; 6%*	77; 6%*	321; 0%

Table S3: Numerical evaluation of microsphere number per reaction for both targets *YE* and *TG*. MFI of the positive samples (MFI+), MFI of the NTCs (MFI-) and height of the signal-to-noise ratio (SNR); one more criterion taking into account the percentage of samples below the set count of 50 was added into consideration (below 50). Weight of each criterion: MFI+ 3, MFI- 2, SNR 1 and below 50 3. The lowest weighted mean represents the most sufficient condition (highlighted in yellow).

**Microsphere input per reaction**	***YE* MFI +**	***YE* MFI -**	***TG* MFI +**	***TG* MFI -**	***YE* SNR**	***TG* SNR**	**Below 50**	**Weighted mean**
**350**	5	4	3	5.5	5	6	5.5	**4.70**
**750**	4	4	4	3	4	3.5	5.5	**4.13**
**1000**	1.5	4	2	5.5	2	3.5	5.5	**3.43**
**1500**	1.5	4	1	7	2	6	5.5	**3.60**
**2500**	3	2	5	2	2	1.5	2.5	**2.87**
**3500**	6	7	6	4	7	6	2.5	**5.23**
**5000**	7	1	7	1	6	1.5	1	**3.77**

Figure S7: Signal-to-noise ratios of seven tested fluorescent reporter dyes. YE = *Y. enterocolitica;* TG = *T. gondii*.

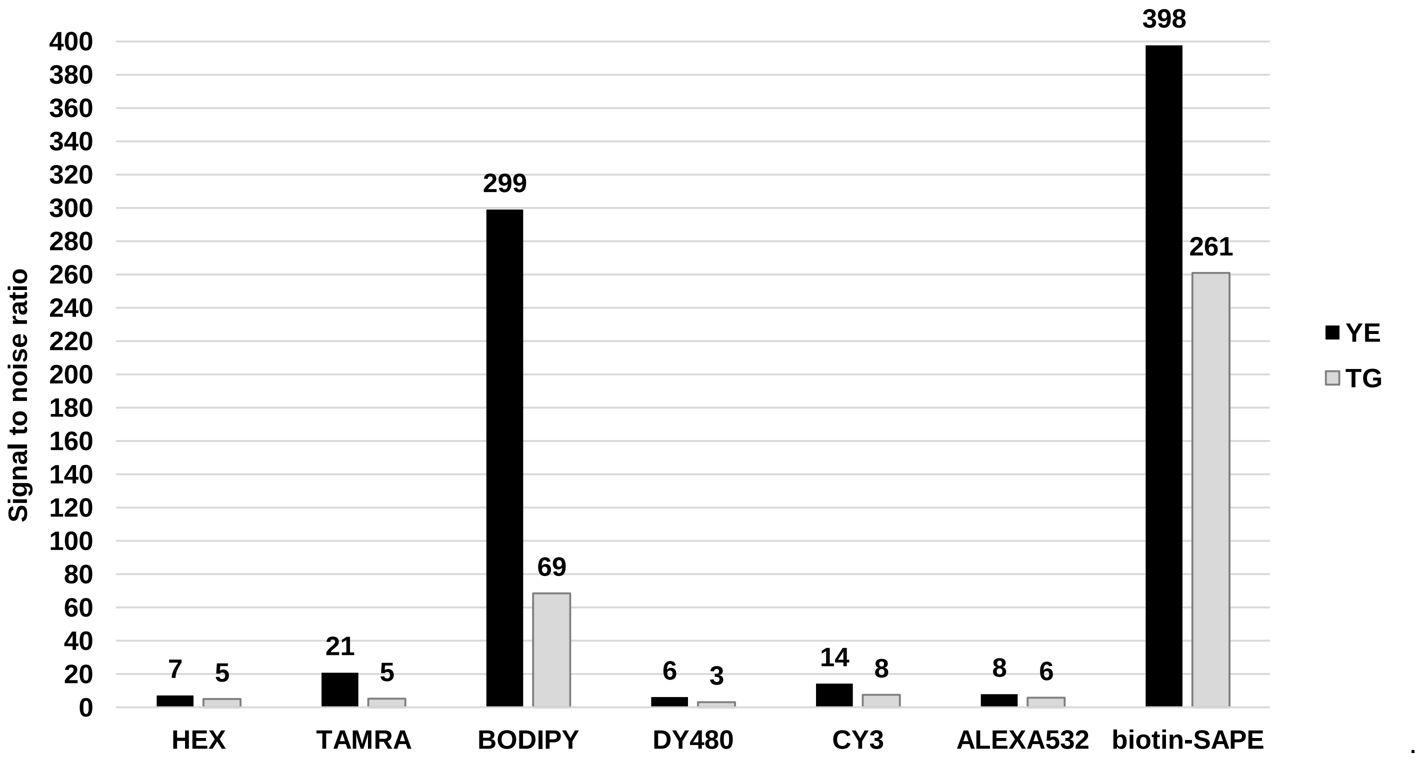

Figure S8: Fluorescent efficiency of biotin-16-dCTPs used for sequence labeling during PCR amplification using SAPE reporter dye. From the values depicted in the graph were signal-to-noise ratios reaching 31 in YE and 26 in TG were calculated. YE = *Y. enterocolitica;* TG = *T. gondii*; plus sign in legend = positive sample; minus sign = NTC.

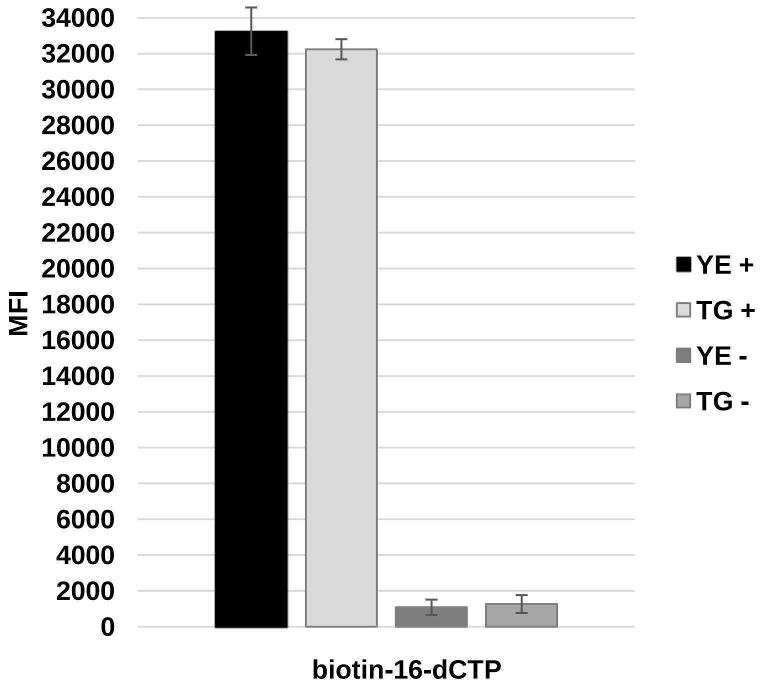

Figure S9: Signal-to-noise ratios of bacterial and parasitic systems tested in an 11-plex foodborne pathogen panel utilizing the BODIPY-REV primer. IAC was exluded from the graph since its signal-to-noise ratio is 1 (the same values in positive samples and NTCs). In graph: TSp = *Trichinella spiralis;* TSa = *Taenia saginata*; EC = *Escherischia coli*; YE = *Y. enterocolitica;* LM = *Listeria monocytogenes;* CJ = *Campylobacter jejuni*; GI = *Giardia intestinalis* generic; GIa = *Giardia intestinalis* assemblage A; TG = *T. gondii*; SE = *Salmonella enterica;* IAC = internal amplification control.

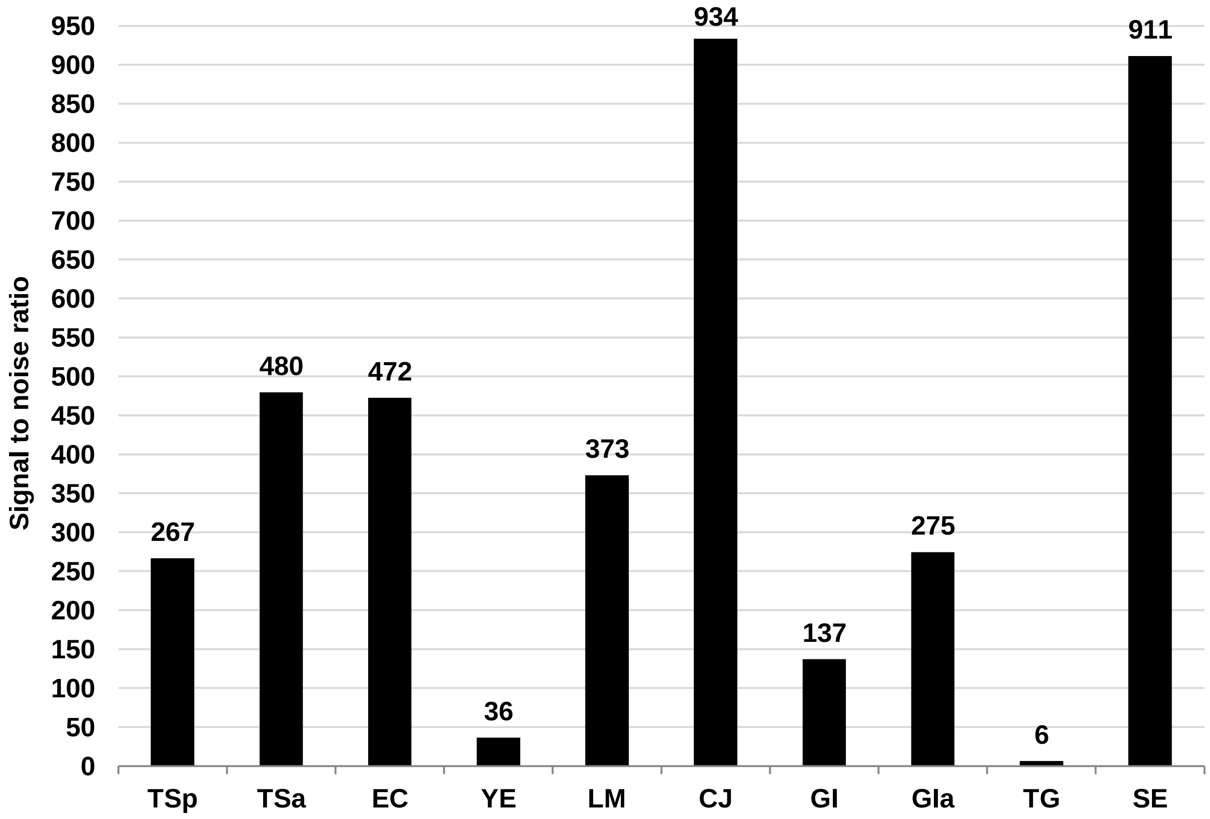

Supplement: Supplementary file 1 — Supplementary Information [file 41598_2019_40035_MOESM1_ESM.pdf]
